# Influence of the Degree of Cure in the Bulk Properties of Graphite Nanoplatelets Nanocomposites Printed via Stereolithography

**DOI:** 10.3390/polym12051103

**Published:** 2020-05-12

**Authors:** Alberto S. De León, Sergio I. Molina

**Affiliations:** Departamento Ciencia de los Materiales, I. M. y Q. I., IMEYMAT, Facultad de Ciencias, Universidad de Cádiz, Campus Río San Pedro, s/n, 11510 Puerto Real (Cádiz), Spain; sergio.molina@uca.es

**Keywords:** 3D printing, stereolithography, nanocomposites, correlation of bulk and molecular properties, physical methods of analysis, mechanical properties, electrical properties

## Abstract

In this work, we report on the fabrication via stereolithography (SLA) of acrylic-based nanocomposites using graphite nanoplatelets (GNPs) as an additive. GNPs are able to absorb UV–Vis radiation, thus blocking partial or totally the light path of the SLA laser. Based on this, we identified a range of GNP concentrations below 2.5 wt %, where nanocomposites can be successfully printed. We show that, even though GNP is well-dispersed along the polymeric matrix, nanocomposites presented lower degrees of cure and therefore worse mechanical properties when compared with pristine resin. However, a post-processing at 60 °C with UV light for 1 h eliminates this difference in the degree of cure, reaching values above 90% in all cases. In these conditions, the tensile strength is enhanced for 0.5 wt % GNP nanocomposites, while the stiffness is increased for 0.5–1.0 wt % GNP nanocomposites. Finally, we also demonstrate that 2.5 wt % GNP nanocomposites possess characteristic properties of semiconductors, which allows them to be used as electrostatic dispersion materials.

## 1. Introduction

Polymer matrix nanocomposites are emerging as cheaper, tougher and lighter materials for a wide variety of applications including energy storage, biomedicine or aerospace and automotive industries [1]. They consist of a nanomaterial such as fibers, particles or 2D materials embedded within a polymeric matrix. When these compounds are combined properly (i.e., a good dispersion of the filler in order to maximize the interfacial area between the filler and the matrix), nanocomposites present enhanced functional and structural properties [2].

Carbon-based additives including carbon fiber, carbon nanotubes, graphene and graphite nanoplatelets (GNPs) have become very popular because of their good mechanical and thermal properties [3,4,5]. In particular, GNPs have been used in different matrixes at the industrial scale due to their inexpensive cost in a different number of matrixes to increase their stiffness. However, their elasticity and tensile strength generally decrease due to their poor compatibility with polymeric matrixes. This effect has been observed in nanocomposites containing polyethylene [6], polyamide [7], polyurethane [8] and epoxy resins [9]. Nonetheless, an enhancement in tensile strength has also been reported when using polylactic acid [10] or thermoplastic elastomers [11] for GNP contents up to 10 and 20 wt %, respectively. Other authors propose chemical strategies to modify GNPs in order to achieve a better compatibility with the matrix and increase the tensile strength of the material [12,13]. The importance of the size of GNPs on the influence of mechanical properties has also been evidenced. In general, it is observed that GNPs with longer lateral dimensions lead to higher stiffness values together with a decrease in tensile strength [8,14]. Moreover, when the GNP content is above a certain amount (i.e., the percolation limit), its incorporation into a nanocomposite increases the electrical and thermal conductivity by several orders of magnitude [15].

Recently, stereolithography (SLA) has emerged as a promising alternative to fabricate carbon-based, polymer matrix nanocomposites [16,17]. SLA is an additive manufacturing technique which consists of the photopolymerization of a liquid precursor using a light source, typically in the UV range, which solidifies the precursor in a layer-by-layer fashion with a resolution in the order of microns [18]. Compared with traditional manufacturing techniques, SLA does not require any mold but only a CAD model with the information of the object to be printed. This allows to perform quick modifications to respond to the constantly changing necessities of the industry, which means saving time and costs in the production [19]. Commercially available resins for SLA are typically made of a combination of multifunctional monomers, diluents and a photoinitiator. Although there are some epoxy-based resin precursors, most widespread SLA precursors are of an acrylic nature [20]. Several groups have successfully modified these precursors with different nanofillers in the design of complex, lightweight nanocomposites for different applications such as microfluidic devices [21], metamaterials [22] or scaffolds for stem cells [23].

The fabrication of carbon-based nanocomposites via SLA allows to obtain tailorable devices with complex shapes, excellent accuracy with a micrometric resolution and enhanced mechanical and electrical properties [24,25]. This enables them as potential candidates for different applications such as dissipative materials [26] or sensors [27]. However, carbon-based additives absorb and scatter the UV light of the laser during the printing process, leading to materials with decreased mechanical properties, so the nanocomposite resin precursor must be carefully prepared [28]. These considerations are critical for the proper design of new materials, since understanding the curing process during 3D printing and its correlation with the macroscopic properties are critical aspects to control the quality of the final printed parts [29].

In this work, we use SLA as an alternative to classic manufacturing techniques to fabricate acrylic-based nanocomposites loaded with GNPs. We study the influence of the additive in the curing process of the resin via a spectroscopic and calorimetric analysis and show how this is related to the overall structural performance by studying their mechanical properties. Finally, we show their potential application as functional materials by evaluating their electrical properties.

## 2. Materials and Methods 

### 2.1. Materials

Form Clear v2 (proprietary mixture of a photoinitiator and of acrylic monomers and oligomers) was purchased from Formlabs. The GNP (avanGRP-40, dimensions 10 µm × 2 µm × 20 nm) was purchased from Avanzare (Navarrete, Spain). Isopropyl alcohol (IPA) was purchased from Scharlab (Sentmenat, Spain). All products were used as received.

### 2.2. Fabrication of Nanocomposites via Stereolithography

Nanocomposite precursors were prepared by mixing the photosensitive resin with different GNP contents (0.5–5 wt %) under high shear mixing and subsequent degasification under vacuum following a previously established protocol [30]. Precursors were then poured into a tank and samples were 3D-printed in a stereolithography printer Form 1+ (Formlabs) using a 405 nm laser with an output power of 120 mW and a spot size of 140 µm. This wavelength matches with one of the local maximum absorbances of the photosensitive resin (see Figure S1 in the Supporting Information for more details). Dogbone specimens according to ASTM D638 (type 1BA) and flat discs of 1–3 mm thickness and 65 mm diameter for measuring the electrical conductivity were printed using a layer height of 200 µm. Samples were detached from the printer platform and were washed with IPA for at least 15 min. Post-processing of the samples was performed for 60 min in a UV chamber (FormCure, Formlabs) previously heated at 60 °C with a light source of 405 nm and power of 1.25 mW/cm^2^. Illustrative examples of the printed specimens and a complex structure can be found in Figure S2 in the Supporting Information.

### 2.3. Material Characterization

Viscosity measurements were performed in a MCR 301 rheometer (Anton Paar GmbH, Graz, Austria). Steady state shear rate sweeps were performed using a 25 mm plate–plate geometry in an interval of 1–1000 s^−1^. At least 3 independent samples were measured. Results were averaged and standard deviations were presented as error bars. X-ray diffraction (XRD) of the samples was measured using a Bruker D8 ADVANCE (Breika, MA, USA) using a Cu Kα radiation source operated at a voltage of 40 kV with a scanning range of 5–80 deg. Raman spectroscopy experiments were performed in an alpha300 system (WITec, Ulm, Germany) coupled to an optical microscope equipped with a piezo scanner (P-500, Physik Instrumente, Hamburg, Germany) and a Nikon 50x objective (NA = 0.6). A linearly polarized laser (λ = 785 nm) was focused onto the sample with a polarization angle of 0° and no analyzer in the light path. The Raman scattered light was detected on a thermoelectrically cooled CCD detector (DU401A-BV, Andor, Belfast, UK) with an integration time of 5 s. Analysis was performed with WiTec Project FOUR 4.1. Single spectra were taken in at least 3 different regions of the samples and results were averaged. Differential scanning calorimetry (DSC) experiments were performed in a Q20 (TA Instruments, New Castle, DE, USA). Temperature sweeps were performed from room temperature (25 °C) to 250 °C at a rate of 10 °C/min to evaluate the residual cure of the resins. A subsequent cooling and heating sweep at 10 °C/min was performed to confirm all the resin was cured in the first sweep. Thermograms were analyzed using the TA Universal Analysis software. Tensile testing of samples was performed in a universal testing machine (Shimadzu, Japan) at a constant speed of 1 mm/min according to ASTM D638. At least 5 specimens were tested and the Young’s modulus, tensile strength and elongation at break values were dissected for each one of the measured specimens. Young’s modulus was determined as the slope between 0.05% and 1.0% strain in the stress–strain plots. Tensile strength was obtained as the maximum stress value in the curve. Elongation at break was obtained as the strain value in the rupture point (maximum value in the X axis). Results were averaged and standard deviations were presented as error bars. Electrical conductivity was measured according to ASTM D257 in a Keithley 6517B electrometer (Keithley, Cleveland, OH, USA) applying a voltage of 500 V. At least 3 samples were measured and results were averaged with standard deviations presented as error bars. Sheet resistance was calculated as the bulk resistivity divided by the thickness of the sample. UV–Vis spectra were recorded in a Varian Cary 50 Conc spectrophotometer. Surface analysis was performed using a scanning electron microscope (SEM) FEI-Quanta 200 3D (Thermo Fisher, Waltham, MA, USA). Samples were coated prior to scanning with a layer of Au in a SCD 004 Sputter Coater (BAL-TEC, Balzers, Lichtenstein).

## 3. Results and Discussion

### 3.1. Fabrication and Compositional Characterization of the Nanocomposites

CAD files were loaded into the SLA software and different objects were printed using the nanocomposite precursors containing 0.5–5.0 wt % GNP. It was observed that the printing of resins containing 0.5–2.5 wt % GNP was successful, while resins containing 3.0–5.0 wt % GNP led to highly irregular objects. A possible reason for this is that the viscosities of the nanocomposite precursors are different. During the SLA process, the tank is tilted after one layer is printed to homogenize the resin precursor before printing the next layer. Too viscous resins have limited capacity to flow during this step, which leads to GNPs local accumulation that interferes with the laser path [31].

**Figure 1:**
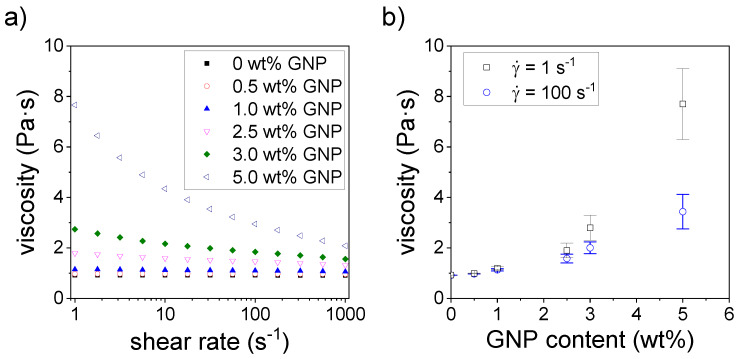
(**a**) Characteristic curve showing shear rate dependency of viscosity for nanocomposite precursors containing different amounts of graphite nanoplatelets (GNPs); (**b**) average viscosity values of nanocomposite precursors at the shear rates of 1 and 100 s^−1^ as a function of the GNPs.

Figure 1a shows the dependency of viscosity with the shear rate. We observe that resins containing GNP up to 1.0 wt % are shear rate-independent, i.e., they behave like ideal, Newtonian fluids. The average viscosity values vary from 0.919 Pa·s for pristine resins to 1.13 Pa·s for resins containing 1.0 wt % GNP. For higher GNP contents, we observe that the viscosity shows a typical shear thinning behavior, increasing when the shear rate is decreased. In our system, it seems that for values above 2 Pa·s, the viscosity is too high to allow a homogeneous flow of the resin, i.e., printing with good quality. To gain further insight, the effect of GNPs on the viscosity at different shear rates was plotted in Figure 1b. Since some of the resin precursors are not Newtonian, we chose to compare the viscosity at the shear rates of 1 and 100 s^−1^. We observe an exponential increase in the viscosity with a GNP content for both shear rates, indicating that GNP has a strong influence on the rheological behavior of the nanocomposite’s precursor. For contents up to 1.0 wt % GNP, the influence on the viscosity is not too high and the precursor still behaves as a Newtonian fluid, therefore, we assume that in these conditions, GNP flows together with the resin in a homogeneous way, which allows defect-free printing via SLA. However, higher GNP contents not only lead to shear thinning behavior but also increase the viscosity significantly, probably due to the aggregation of GNP caused by its lack of interaction with the polymeric matrix [32]. The agglomeration of GNP may impede the resin to flow properly, and also partially block the light path of the laser, causing defective printing. An exponential increase in viscosity has also been observed when using other inorganic fillers with a high aspect ratio such as montmorillonite (MMT) [33], carbon nanotubes [34] or hydroxyapatite [35]. Weng et al. [33] reported that SLA printing was not possible for MMT concentrations where a strong shear thinning behavior was observed, similar to our results. When the shear rate is high enough, it can cause the disaggregation and orientation of 2D nanomaterials, such as GNP or MMT. However, if the shear rate is too low, nanoparticles aggregate and impede the proper flow of the resin. This effect is stronger when the concentration of the additive is increased. They claimed that defective printing is caused by two factors: first, the high viscosity and non-Newtonian behavior leads to an insufficient flow in the tank; second, the presence of these 2D nanomaterials may absorb or scatter the laser UV radiation to a relatively large extent, which decreases the light dose received by the photocurable resin. The combination of these two features may lead to an incomplete curing, reduction in the mechanical properties and defects in the printed objects, as it was observed in our case for GNP contents above 2.5 wt %.

**Figure 2:**
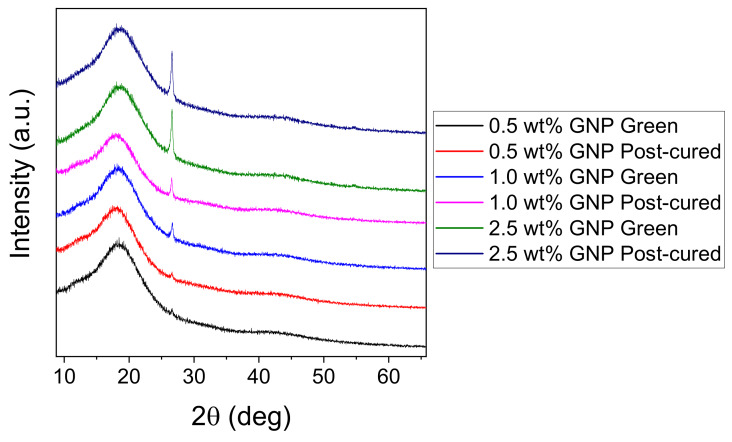
XRD characteristic curves of green and post-cured nanocomposites containing 0–2.5 wt % GNP.

Hence, nanocomposites having a maximum content of 2.5 wt % GNP were printed and thoroughly washed with IPA (green samples). Then, at least five samples of each concentration were post-processed inside a UV chamber at 60 °C for 60 min (post-cured samples). The stability of the GNP before and after post-curing was studied by XRD. Figure 2 shows a broad peak around 18.5 deg corresponding to the acrylic resin matrix, due to its amorphous nature. We can also see a sharp peak at 26.5 deg, characteristic of the crystalline structure of graphite [30], which increases gradually with the GNP content in the resin. These analyses were performed at different regions of a printed specimen and no significant differences in the GNP peak intensity were observed indicating that, at least during the 3D printing process times, GNP is homogeneously dispersed in the resin medium. The similar intensity before and after the post-curing process indicates that, as expected, post-processing does not alter the morphology or the structural stability of the GNP in the resin.

**Figure 3:**
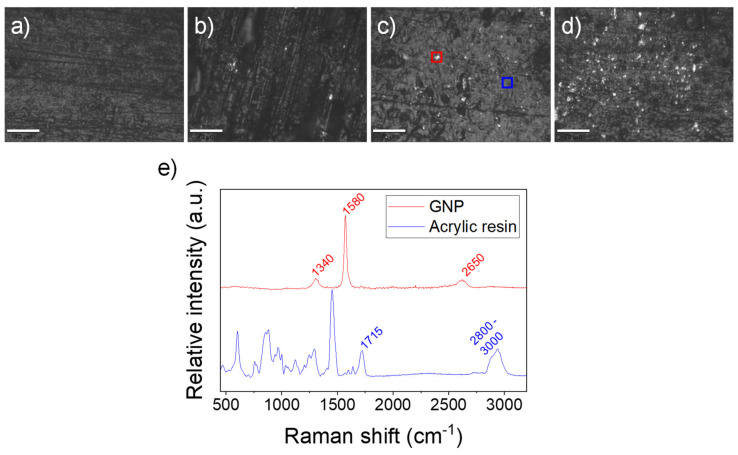
Optical micrographs of printed resins containing (**a**) no GNP; (**b**) 0.5 wt % GNP; (**c**) 1.0 wt % GNP and (**d**) 2.5 wt % GNP; (**e**) characteristic Raman single-spectra taken in two different spots of a sample containing 1.0 wt % GNP, marked in Figure 3c. Scale bar: 70 µm.

The characterization of the composites was done using Raman microscopy. This device consists of a Raman spectrometer coupled to an optical microscope where the surface of the sample can be imaged for an in situ spectroscopic analysis. Figure 3a–d and Figure S3 show the optical microscopy images of the surface of the different nanocomposites, where scattered bright particles on a matte background can be observed. We attribute these to the GNP and the acrylic resin, respectively. These bright particles are not observed in Figure 3a, where there is no GNP. According to the fabricant, the theoretical GNP size is 2 × 10 × 0.020 µm, so this seems a very plausible hypothesis. As demonstrated in Figure 2, the GNP is a crystalline material, while the acrylic resin is highly amorphous, so we state that they can present very different optical behavior under an inverted light microscope.

We analyzed the Raman spectra in these regions (marked as red and blue squares in Figure 3c and, as expected, different spectra were obtained. The red color spectrum of Figure 3e shows the characteristic D, G and 2D peaks at 1340, 1580 and 2650 cm^−1^, respectively, when Raman is performed in the bright particles. The intensity of the G peak (1580 cm^−1^) is higher than the 2D one, which is characteristic of graphitic structures (I_2D_/I_G_ < 1) [36]. This supports that mechanical agitation can effectively disperse the GNP in a polymeric matrix but does not achieve its exfoliation into graphene [37]. When a Raman spectrum is acquired elsewhere, a characteristic footprint of an acrylic polymer is observed. The blue color spectrum of Figure 3e shows the characteristic peak at 1715 cm^−1^ corresponding to the C=O stretching in the acrylate groups as well as the band at 2800–3000 cm^−1^ corresponding to the C–H stretching of the CH_3_ and CH_2_ groups in the polymer main chains [38]. As complementary measurements, we analyzed the nanocomposites using SEM (Figure S4 in the Supporting Information). However, in this case, we found it more difficult to distinguish the GNP flakes from the roughness of the surface of the acrylic matrix. Thus, we concluded that Raman microscopy is a better tool to identify the GNP spatial distribution in a polymeric matrix.

### 3.2. Quantitative Analysis of the Degree of Cure of the Nanocomposites

**Figure 4:**
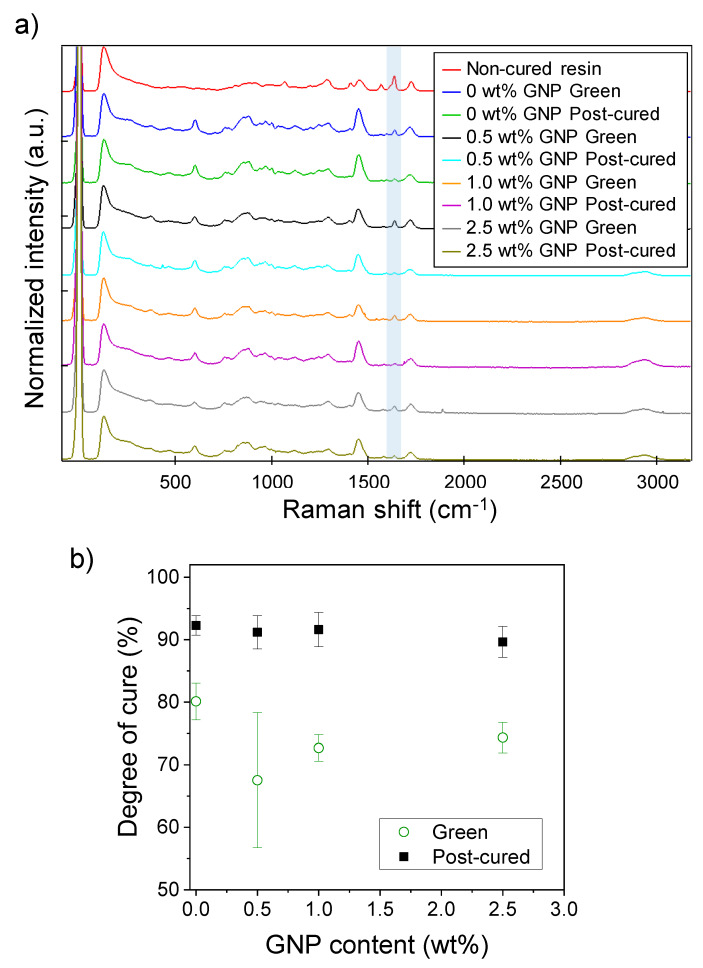
(**a**) Characteristic Raman scattering spectra of non-cured, green and post-cured resins containing 0–2.5 wt % GNP. Peaks at 1636 cm^−1^ corresponding to the CH_2_=CH–R stretching of the acrylate groups are highlighted in blue; (**b**) amount of resin cured obtained from the Raman spectroscopy results as a function of the GNP content for the green and post-cured samples.

A detailed analysis of the Raman spectra of the different nanocomposites was performed in order to obtain quantitative information of the degree of cure. Figure 4a shows the characteristic Raman spectra for non-cured liquid resin and green and post-cured nanocomposites containing 0, 0.5, 1.0 and 2.5 wt % GNP. To ensure the reproducibility of the results, at least three spectra were taken in different spots and the results were averaged. The spectra were normalized to the C=O peak at 1715 cm^−1^, since it does not participate in the curing process. It can be observed that there is a decrease in the intensity of the peak at 1636 cm^−1^, which corresponds to the CH_2_=CH–R stretching of the acrylate monomers, for the green and post-cured samples when compared with the non-cured resin, indicating the polymerization of the acrylic monomers. However, a small residual peak is always observed, which indicates that curing is not fully complete even after the post-processing with UV light. We quantified the areas corresponding to this peak at 1636 cm^−1^ and we used Equation (1) for estimating the degree of cure of the resin. $A_{1636cm^{-1},\;non-cured}$ is the area of the non-cured resin (red curve in Figure 4a), while $A_{1636cm^{-1}}$ corresponds to the area of the different nanocomposites after printing or post-curing:

(1) $$Degree\;of\;cure\;\left(\%\right)=\left(1-\frac{A_{1636cm^{-1}}}{A_{1636cm^{-1},\;non-cured}}\right)\times 100$$

The degree of cure depending on the GNP content is presented in Figure 4b. It can be observed that the degree of cure decreases for the green samples regardless of the GNP content. In the absence of GNPs, the green samples present a degree of cure of 80.1%, while this value decreases down to 67.5–74.3% for GNP contents ranging from 0.5 to 2.5 wt %. This seems to indicate that GNP partially blocks the activation and propagation of the monomers that participate in the polymerization of the resin. However, after the post-curing process, the degree of cure is increased up to 89.6–92.3% for all the studied cases, including the acrylic resin containing no GNP. These results suggest that a degree of cure higher than 60% allows the satisfactory fabrication of self-standing objects via SLA, although the cure kinetics were slowed down. Then, since the post-curing process is performed inside a UV chamber, the polymerization of the remaining acrylate monomers occurs in a more homogeneous way instead of locally, as it happens during the SLA process. This leads to materials with practically the same degree of cure, regardless of the GNP content used. It is important to remind that 3D printing was not possible for contents above 2.5 wt % GNP. However, these results suggest that 2.5 wt % GNP nanocomposites can be successfully post-cured, reaching practically the same final degree of cure as the pristine resin.

The degree of cure has been measured via spectroscopic techniques (typically FTIR) for classical fabrication techniques of resins [39] and acrylic resins with potential use in SLA [40], where a degree of cure up to 60% has been observed. However, to the best of our knowledge, so far only Martin et al. [41] have recently studied the kinetics and degrees of cure for epoxy and acrylic resins via Raman spectroscopy in SLA. In their research, the acrylic precursor needed several seconds to achieve full polymerization when using a laser power of 100 mW. This supports our results, since we do not have full conversion for a similar laser output power and irradiation times under 1 s. Moreover, in our case we studied the influence of a nanofiller and UV post-processing on the degree of cure and this would be the first time that it is quantified for nanocomposites prepared by SLA.

**Figure 5:**
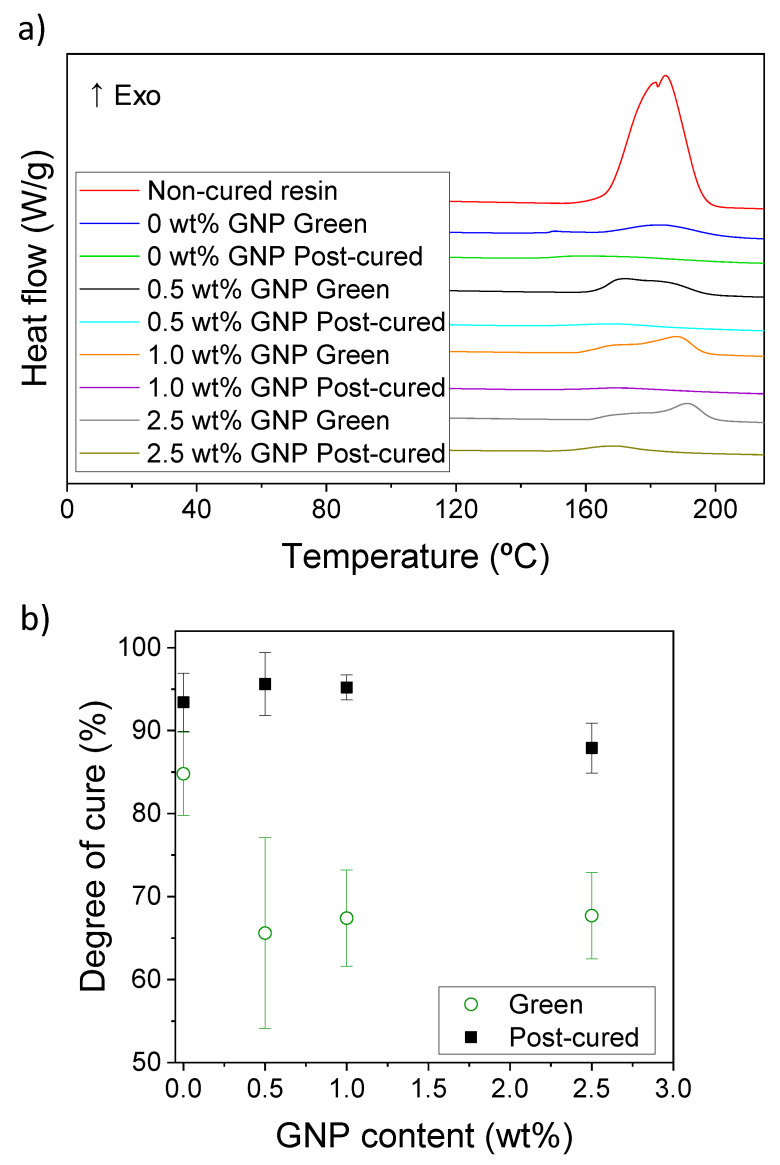
(**a**) Differential scanning calorimetry (DSC) thermograms of the non-cured, green and post-cured resins containing 0–2.5 wt % GNP; (**b**) amount of resin cured obtained from the DSC results as a function of the GNP content for the green and post-cured samples.

In addition to the spectroscopic analyses, the degree of cure was also determined by DSC. DSC is also a widespread technique used for measuring the residual cure of different thermosetting polymers [42], but little research has been performed for the characterization of 3D printed materials via SLA. In our case, as it can be observed in Figure 5a, acrylic resins exhibit an exothermic peak at 170–185 °C, corresponding to the thermal polymerization of the acrylate groups. This peak is particularly clear for non-cured, liquid resin, where we assume all the acrylate groups are still unreacted and its energy value corresponds to the full conversion of the acrylate groups, i.e., the cure enthalpy ($\Delta H_{cure}$). A smaller peak can be observed for the green samples and a practically unnoticeable peak is also detected for the post-cured samples. By quantifying these areas, associated with the residuary cure enthalpy ($\Delta H_{residuary}$), the degree of cure can be calculated in each case according to Equation (2):

(2) $$Degree\;of\;cure\;\left(\%\right)=\left(1-\frac{\Delta H_{residuary}}{\Delta H_{cure}}\right)\times 100$$

Figure 5b summarizes the degree of cure for the green and post-cured nanocomposites for the different GNP amounts used. It can be observed that the presence of GNPs inhibits the curing for the green samples, decreasing the degree of cure from 84.8% for pristine resins to 65.6–67.7% for GNP contents of 0.5–2.5 wt %. This average variation is higher than the one obtained from the Raman analysis (Figure 4b). However, when errors are considered, we observe that the results obtained from the Raman and DSC are similar, these values being overlapped. A similar trend is observed for the post-cured samples. The average degrees of cure ranging from 87.9 to 95.6% are in good agreement with the 89.6–92.3% observed in the Raman spectroscopy. From the DSC results, the resins containing 2.5 wt % GNP show slightly lower values of the degree of cure. We believe this is because the amount of GNP still partially blocks the UV light inside the curing chamber during the post-curing processing.

In any case, we state that the DSC and Raman spectroscopy are complementary techniques and lead to similar results, presented in Table 1. The Raman spectroscopy results seem in general more accurate, since they present smaller standard deviations. Moreover, the data acquisition is much faster in the case of Raman spectroscopy, where integration times of 5 s are used, while for a single sweep in DSC we need more than 1 h, so we consider that Raman spectroscopy is probably a better technique for the quantification of the degree of cure. In any case, as a general conclusion, we demonstrated that GNPs presence is able to partially impede the polymerization of the acrylic resin during SLA, causing a decrease of 10%–20% in the degree of cure of the resin. However, this difference disappears (i.e., the values are not statistically significant) after a post-curing process at 60 °C for 1 h inside a UV chamber, which led to a practically fully cured resin (90%–95%). Non-cured monomers, even after the post-curing process, could be explained because of steric hindrance. As the polymeric 3D network is formed, monomers find more difficulties when trying to diffuse across this network [43].

### 3.3. Mechanical and Electrical Properties of the Nanocomposites

**Figure 6:**
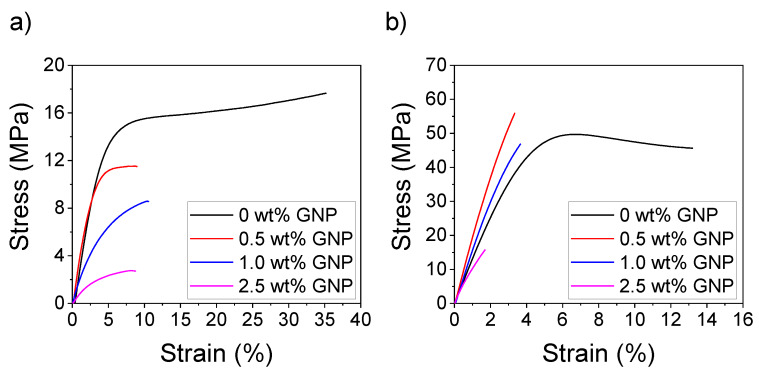
Representative strain–stress curves for the (**a**) green and (**b**) post-cured nanocomposites containing 0–2.5 wt % GNP.

We performed tensile testing experiments to study the influence of GNP on the acrylic matrix and evaluate the overall mechanical performance of the nanocomposites. Figure 6a shows the representative strain–stress curves for the green nanocomposites. We observe that the mechanical performance of the nanocomposites is, in general, worse than the pristine resin. The tensile strength and Young’s modulus decrease proportionally when the GNP content is increased and the elongation at break highly decreases from 35% to 7%–9% strain.

After post-curing, the mechanical properties sensibly change. Figure 6b shows that the Young’s modulus and tensile strength increase while the elongation at break decreases for all the studied cases because new polymeric chains and crosslinking points are formed during this process. The influence of the post-curing on the increase in the strength of the resins printed in SLA without any filler has already been studied. It is generally assumed that subsequent exposure to UV light after printing will lead to the polymerization of the residual monomers, increasing the number of covalent bonds which will provide the material with enhanced strength in spite of the loss in elasticity [44].

**Figure 7:**
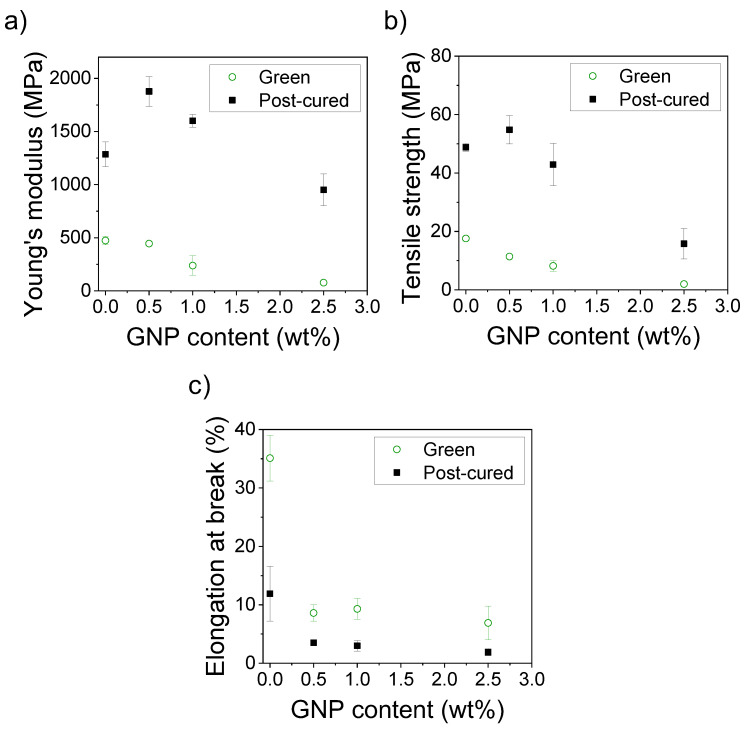
Average (**a**) Young’s modulus; (**b**) tensile strength and (**c**) elongation at break for the green and post-cured nanocomposites containing 0–2.5 wt % GNP.

For the post-cured nanocomposites, we find that the Young’s modulus and tensile strength significantly increase from 1280 MPa and 48.8 MPa to 1880 MPa and 54.8 MPa, respectively, for the 0.5 wt % GNP nanocomposites. The Young’s modulus also increases up to 1600 MPa for the 1.0 wt % GNP nanocomposites. However, the mechanical properties are not enhanced for the resins containing 2.5 wt % GNP. We found that post-curing times higher than one hour did not lead to any significant variation in the mechanical properties of the nanocomposites. A summary of the variation of these mechanical properties can be found in Figure 7.

Hence, we state that it is critical to consider the degree of cure after SLA for printed nanocomposites, since the presence of GNPs impedes the polymerization of the acrylic monomers, leading to lower degrees of cure after the printing process. In terms of the molecular structure, these nanocomposites possess less densely packed 3D networks which present worse overall mechanical performance when compared with the pristine resin. After the post-processing in the UV chamber, the degree of cure increases in these cases, practically reaching the full conversion of the monomers (DC > 90%). This implies that the polymeric network formed is similar and we can attribute the changes observed in the mechanical properties to the presence of GNPs. Actually, we observe enhanced mechanical properties for the post-cured nanocomposites in the elastic regime. After post-processing, the elongation at break is still sensibly lower due to the lack of interactions between the GNP and acrylic resin due to the inorganic nature of the filler and the organic nature of the acrylic resin that acts as a matrix. This explanation can also be valid for the nanocomposites containing 2.5 wt % GNP, where the lack of compatibility between the GNP and the polymeric matrix leads to a weak and fragile material.

There are some previous reports discussing the influence of the filler in the mechanical properties of materials manufactured by SLA. In agreement with our results, a decrease in the strength and hardness has been reported for nanocomposites containing GNPs without any post-processing after printing [28]. However, Gonzalez et al. [34] have reported an increase in the stiffness for acrylic resins loaded with carbon nanotubes up to 0.3 wt %, after a post-processing with UV light. Above this amount, they reported a decrease in the stiffness. Other authors show increase in the tensile strength and stiffness when using silica nanoparticles as reinforcements up to 5 wt %. However, they observed only increased stiffness when they used 1D and 2D materials, evidencing that not only the chemical nature but also the morphology plays a crucial role in the mechanical properties of these materials [33]. For epoxy-based nanocomposites manufactured by classical techniques, in most of the cases, a general trend is observed when GNPs are used as a filler. For GNP contents in the range of 1–5 wt %, the stiffness increases while the tensile strength decreases or, in the best of the cases, remains constant [9,12,14,45]. On the other hand, Zhang et al. [13] report an increase in the stiffness and tensile strength for contents up to 0.5 wt %. This is in good agreement with our results for the post-cured nanocomposites, and seems to support that 0.5 wt % might be the optimal amount to have nanocomposites with an enhanced strength and stiffness. In summary, we believe these results evidence the importance of post-processing for printable GNP-based nanocomposites with enhanced structural properties.

**Figure 8:**
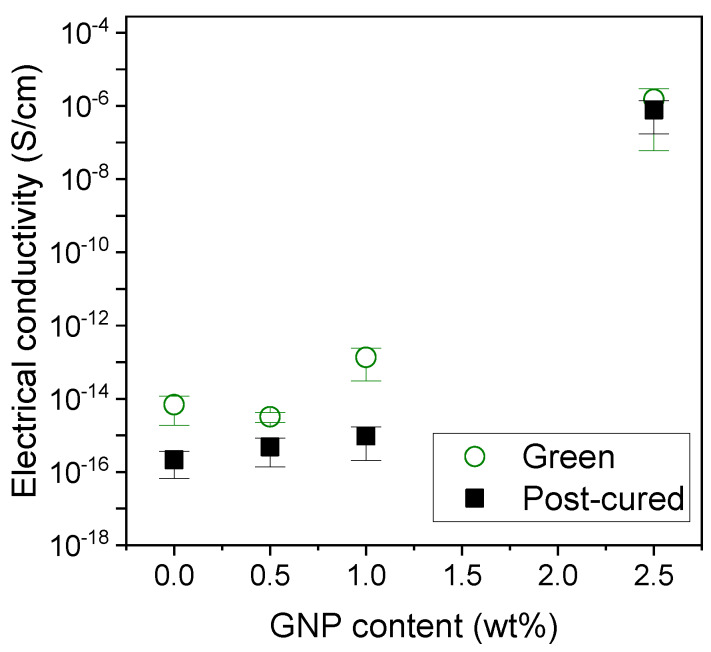
Electrical conductivity for the green and post-cured nanocomposites containing 0–2.5 wt % GNP.

The electrical properties of the nanocomposites were also measured. Most polymers, including acrylic resins, are well-known to be non-conductive, insulating materials (σ_e_ < 10^−8^ S/cm). As it is shown in Figure 8, the electrical conductivity for the pristine resins are in the range 10^−14^–10^−16^ S/cm, meaning a strong insulating behavior. There were no significant changes for the 0.5 wt % GNP nanocomposites and a slight increase of one order of magnitude was observed for the 1.0 wt % GNP green nanocomposites. In general, a small decrease of approximately one order of magnitude is achieved for the post-cured samples when compared with the green ones. We attribute this to a lower mobility of the electrons along the nanocomposite since the degree of cure is higher and the monomer content has sensibly decreased after the post-processing. In terms of the molecular structure, monomers are much smaller molecules that can move more freely, which could somehow enhance the electron mobility. Interestingly, a huge increase of nine orders of magnitude is observed when the GNP content is increased up to 2.5 wt %, reaching conductivity values in the range of 10^−6^ S/cm. In this case, we do not see considerable differences between the green and post-cured samples. We believe that in this case, electrons can find more easily their path along the GNP additives and the degree of cure is not relevant for their mobility. However, the percolation limit does not seem to be reached yet, since these values correspond to a semiconductor rather than to a conductive material [10]. This increase is in good agreement with other nanocomposites found in the literature. For instance, Bakir et al. [46] have recently reported that the percolation limit for nanocomposites containing GNPs is around 4–6 wt %, depending on their lateral size. However, as we showed initially, nanocomposites with GNP contents higher than 2.5 wt % cannot be successfully printed via SLA. This limits the conductivity ranges that we can achieve, and at least in the conditions we used, the electric percolation limit could not be overcome. Nonetheless, the 2.5 wt % GNP nanocomposites present interesting properties, since their sheet resistance value is in the range 10^7^–10^9^ Ohm/sq. This value is characteristic of dissipative materials, which makes it a good material for preventing equipment and devices against electrostatic discharges (ESD) [47]. The pristine resin, 0.5 and 1.0 wt % GNP nanocomposites present sheet resistance values above 10^11^ Ohm/sq, which is characteristic of insulators, but cannot act as ESD materials.

## 4. Conclusions

In this paper, we have successfully printed nanocomposites consisting of an acrylic matrix and GNPs as an additive via SLA. We have established a window of processability of the GNPs content (up to 2.5 wt %) where nanocomposites can be manufactured with a homogeneous distribution of the GNPs in the acrylic matrix. We have quantitatively studied the influence of GNPs in the degree of cure after printing and UV post-processing and correlated these values with the mechanical properties of the nanocomposites. In particular, we observed that a decrease of approximately 10% in the degree of cure had a strong influence on the loss of the mechanical properties for the green samples. However, the stiffness and tensile strength can be enhanced for the post-cured 0.5–1.0 wt % GNP nanocomposites, since in these conditions, all materials possess a similar degree of cure (degree of cure > 90%). On the other hand, nanocomposites containing 2.5 wt % GNP present electrical properties in the range of semiconductors’ dissipative materials (10^7^ Ohm/sq), which enables them as promising candidates for ESD prevention.

## Figures and Tables

**Table 1:** Summary of the degrees of cure determined via the Raman spectroscopy and DSC for the green and post-cured nanocomposites containing 0–2.5 wt % GNP.

GNP Content (wt %)	Degree of Cure (%)			
	Green Samples		Post-cured Samples	
	Raman	DSC	Raman	DSC
**0**	80.1 ± 2.9	84.8 ± 5.0	92.3 ± 1.5	93.4 ± 3.5
**0.5**	67.5 ± 10.8	65.6 ± 11.5	91.2 ± 2.6	95.6 ± 3.8
**1.0**	72.7 ± 2.1	67.4 ± 5.8	91.6 ± 2.7	95.2 ± 1.5
**2.5**	74.3 ± 2.4	67.7 ± 5.2	89.6 ± 2.5	87.9 ± 3.0

## Supplementary Materials

Supporting Information containing Figures S1–S4 is available online at https://www.mdpi.com/2073-4360/12/5/1103/s1.
