# Supplementary material for: Influence of the Degree of Cure in the Bulk Properties of Graphite Nanoplatelets Nanocomposites Printed via Stereolithography
Source: Polymers (Basel). 2020 May 12;12(5):1103. doi: 10.3390/polym12051103 (PMC7285314; doi:10.3390/polym12051103)
Supplement: Supplementary file 1 [file polymers-12-01103-s001.pdf]

SUPPORTING INFORMATION

Influence of the Degree of Cure in the Bulk Properties of Graphite Nanoplatelets Nanocomposites Printed via Stereolithography

Alberto S. de León*, Sergio I. Molina

Dpto. Ciencia de los Materiales, I. M. y Q. I., IMEYMAT, Facultad de Ciencias, Universidad de Cádiz, Campus Río San Pedro, s/n, 11510 Puerto Real (Cádiz), Spain; sergio.molina@uca.es

*corresponding author: alberto.sanzdeleon@uca.es

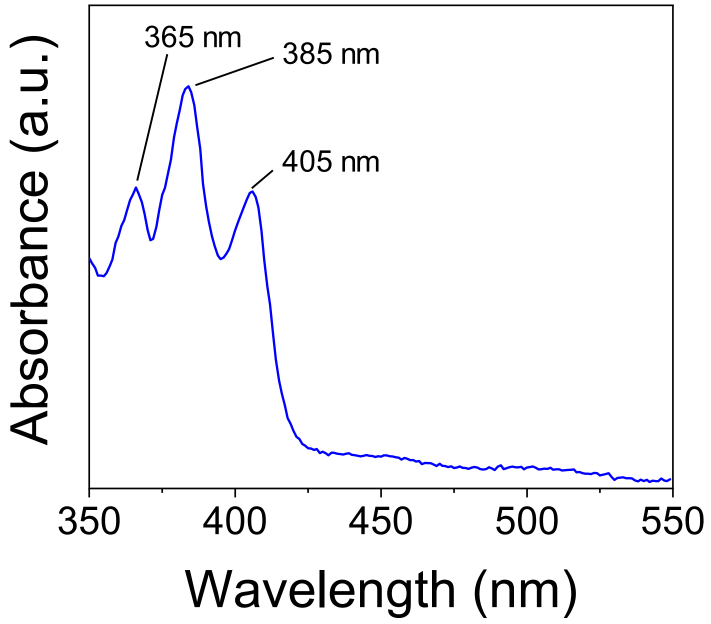

Figure S1. Absorbance spectrum of the liquid resin.

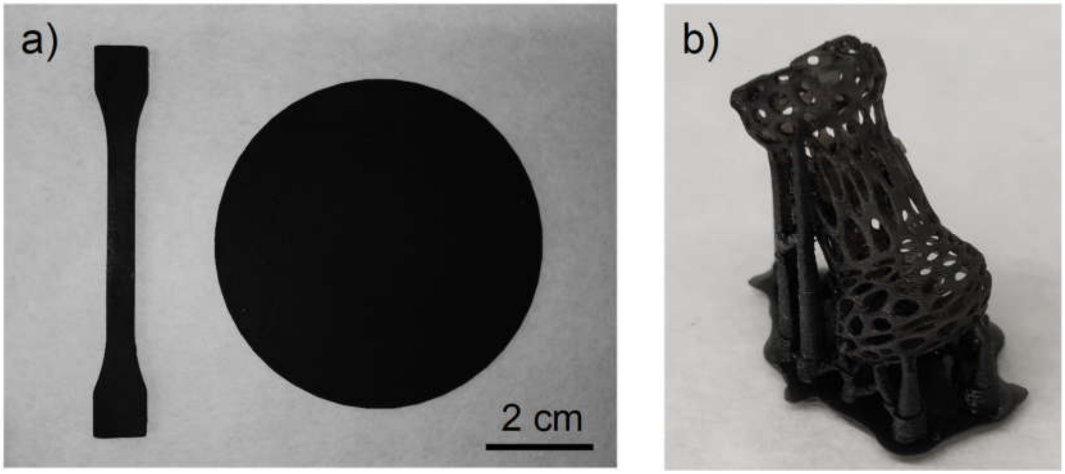

Figure S2. Illustrative images of a) a tensile testing specimen and an electrical conductivity specimen and b) a complex structure of nanocomposites containing 0.5 wt% GNP. Samples were printed with a layer height of 200 µm.

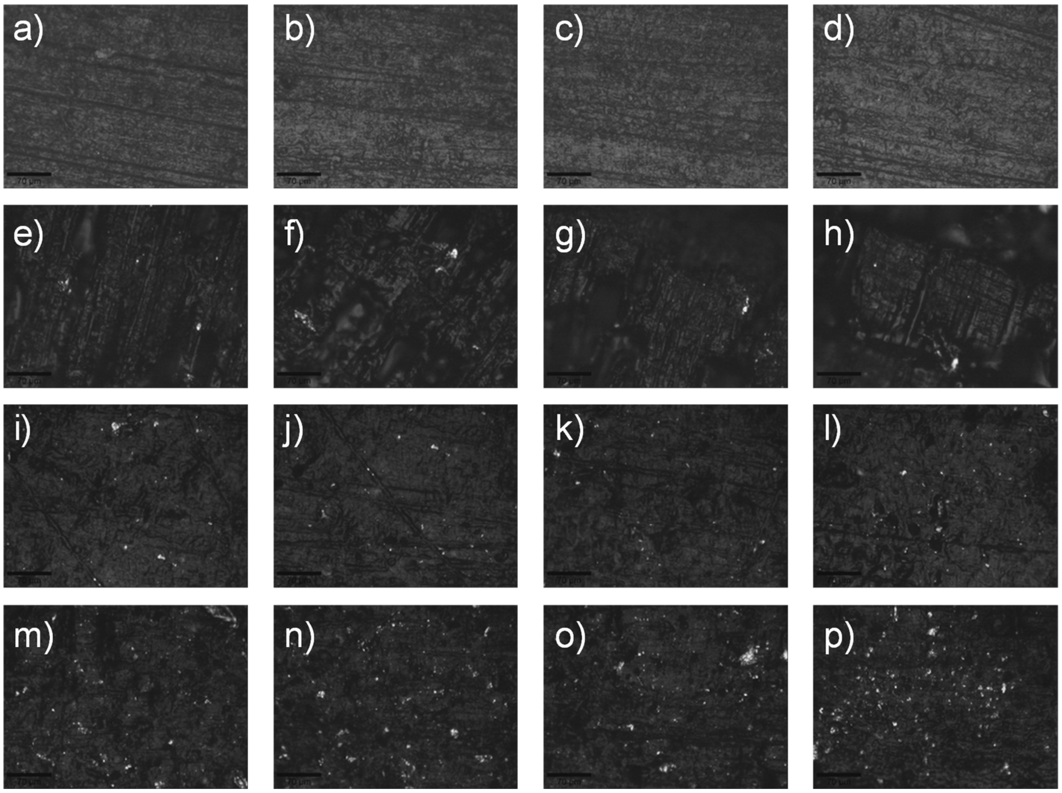

Figure S3. OM micrographs of printed acrylic resins containing a-d) no GNP; e-h) 0.5 wt% GNP; i-l) 1.0 wt% GNP and m-p) 2.5 wt% GNP. Scale bar: 70 µm.

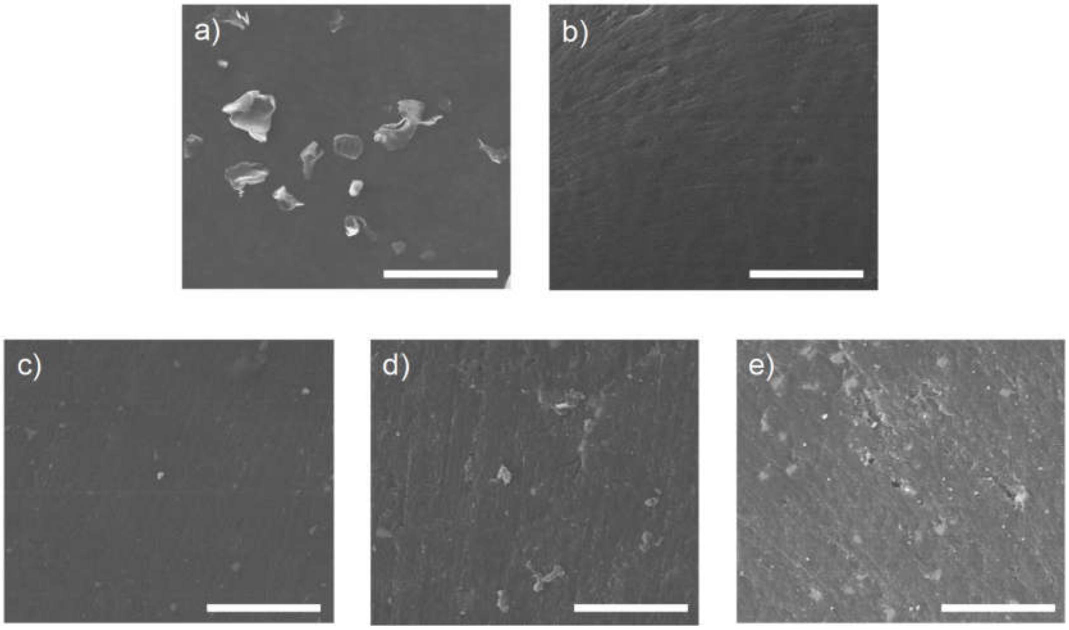

Figure S4. SEM micrographs of a) GNP; printed acrylic resins containing b) no GNP; c) 0.5 wt% GNP; d) 1.0 wt% GNP and e) 2.5 wt% GNP. Scale bar: 50 µm.
